# *In Vivo* Experiments Reveal the Good, the Bad and the Ugly Faces of sFlt-1 in Pregnancy

**DOI:** 10.1371/journal.pone.0110867

**Published:** 2014-11-13

**Authors:** Gabor Szalai, Yi Xu, Roberto Romero, Tinnakorn Chaiworapongsa, Zhonghui Xu, Po Jen Chiang, Hyunyoung Ahn, Birgitta Sundell, Olesya Plazyo, Yang Jiang, Mary Olive, Bing Wang, Suzanne M. Jacques, Faisal Qureshi, Adi L. Tarca, Offer Erez, Zhong Dong, Zoltan Papp, Sonia S. Hassan, Edgar Hernandez-Andrade, Nandor Gabor Than

**Affiliations:** 1 Perinatology Research Branch, Eunice Kennedy Shriver National Institute of Child Health and Human Development, National Institutes of Health, Department of Health and Human Services, Bethesda, MD, and Detroit, MI, United States of America; 2 Department of Obstetrics and Gynecology, Wayne State University School of Medicine, Detroit, MI, United States of America; 3 Department of Pharmacology, Wayne State University School of Medicine, Detroit, MI, United States of America; 4 Department of Pathology, Wayne State University School of Medicine, Detroit, MI, United States of America; 5 Department of Computer Science, Wayne State University, Detroit, MI, United States of America; 6 Center for Molecular Medicine and Genetics, Wayne State University School of Medicine, Detroit, MI, United States of America; 7 Department of Obstetrics and Gynecology, Soroka University Medical Center, School of Medicine, Faculty of Health Sciences, Ben Gurion University of the Negev, Beer Sheva, Israel; 8 Maternity Private Department, Kutvolgyi Clinical Block, Semmelweis University, Budapest, Hungary; 9 Institute of Enzymology, Research Centre for Natural Sciences, Hungarian Academy of Sciences, Budapest, Hungary; University Hospital Basel, Switzerland

## Abstract

**Objective:**

Soluble fms-like tyrosine kinase (sFlt)-1-e15a, a primate-specific sFlt-1-isoform most abundant in the human placenta in preeclampsia, can induce preeclampsia in mice. This study compared the effects of full-length human (h)sFlt-1-e15a with those of truncated mouse (m)sFlt-1(1-3) used in previous preeclampsia studies on pregnancy outcome and clinical symptoms in preeclampsia.

**Methods:**

Mice were injected with adenoviruses or fiber-mutant adenoviruses overexpressing hsFlt-1-e15a, msFlt-1(1-3) or control GFP under the CMV or *CYP19A1* promoters on gestational day 8 (GD8) and GD11. Placentas and pups were delivered by cesarean section, and dams were monitored postpartum. Blood pressure was telemetrically recorded. Urine samples were collected with cystocentesis and examined for albumin/creatinine ratios. Tissue specimens were evaluated for transgene as well as endogenous mFlt-1 and msFlt-1-i13 expression. H&E-, Jones- and PAS-stained kidney sections were histopathologically examined. Placental GFP expression and aortic ring assays were investigated with confocal microscopy.

**Results:**

Mean arterial blood pressure (MAP) was elevated before delivery in hsFlt-1-e15a-treated mice compared to controls (GD18: ΔMAP = 7.8 mmHg, p = 0.009), while ΔMAP was 12.8 mmHg (GD18, p = 0.005) in msFlt-1(1-3)-treated mice. Urine albumin/creatinine ratio was higher in hsFlt-1-e15a-treated mice than in controls (GD18, p = 0.04; PPD8, p = 0.03), and msFlt-1(1-3)-treated mice had marked proteinuria postpartum (PPD8, p = 4×10^−5^). Focal glomerular changes were detected in hsFlt-1-e15a and msFlt-1(1-3)-treated mice. Aortic ring microvessel outgrowth was decreased in hsFlt-1-e15a (p = 0.007) and msFlt-1(1-3)-treated (p = 0.02) mice. Full-length msFlt-1-i13 expression was unique for the placenta. In hsFlt-1-e15a-treated mice, the number of pups (p = 0.046), total weight of living pups (p = 0.04) and maternal weights (p = 0.04) were higher than in controls. These differences were not observed in truncated msFlt-1(1-3)-treated mice.

**Conclusions:**

Truncated msFlt-1(1-3) simulated the preeclampsia-promoting effects of full-length hsFlt-1. MsFlt-1(1-3) had strong effect on maternal endothelium but not on placentas and embryos. In contrast, hsFlt-1-e15a induced preeclampsia-like symptoms; however, it also increased litter size. In accord with the predominant placental expression of hsFlt-1-e15a and msFlt-1-i13, full-length sFlt-1 may have a role in the regulation of embryonic development. These observations point to the difference in the biological effects of full-length and truncated sFlt-1 and the changes in the effect of full-length sFlt-1 during pregnancy, and may have important implications in the management of preeclampsia.

## Introduction

Human pregnancy is a unique state of maternal-fetal immune tolerance [1]–[13], and it was thought to be predominantly an anti-inflammatory state [14]. A growing body of evidence now suggests that three phases can be distinguished at the implantation site with advancing gestation [12], [13]. In early pregnancy, pro-inflammatory mechanisms play key roles in trophoblast invasion and remodeling of maternal spiral arteries, resulting in the adequate blood supply for the placental-fetal unit [15], [16]. This period is followed by an anti-inflammatory state characterized by accelerated fetal growth and development. At the end of pregnancy, pro-inflammatory pathways are key in spontaneous parturition, promoting the activation of the decidua and myometrium, cervical ripening, uterine contractions, and the delivery of the fetus and placenta [17]-[24]. However, the early activation of local pro-inflammatory mechanisms involved in parturition as well as systemic inflammation in the mother are central to the development of most obstetrical syndromes (e.g. preterm birth, preeclampsia or unexplained fetal demise) [20], [25]–[47].

Different phases of the physiological oxygen supply for the feto-placental unit were also identified in humans. A low-oxygen environment is key for the establishment of pregnancy since it favors trophoblastic cell proliferation, placental angiogenesis and embryonic organogenesis [48]. After a transition with the onset of maternal circulation of the placenta at the end of the first trimester, higher oxygen concentration will be available to support rapid fetal development [48]–[53]. However, the early-onset of maternal circulation in the placenta introduces excessive placental oxidative stress, leading to villous regression and miscarriages [48], [51], [53]. Placental oxidative stress later in pregnancy caused by the abnormal remodeling of spiral arteries, placental under-perfusion and fluctuating placental oxygen concentrations is central to the development of fetal growth restriction and preeclampsia [48], [54], [55].

Angiogenesis, a key element in placental and fetal development, is strongly interconnected with inflammation and oxygen signaling [56]–[58]. The placenta is a rich source of pro- and anti-angiogenic factors [59]–[62], and there is a physiological increase in the placental production and systemic availability of pro- and anti-angiogenic molecules with advancing gestation [62]–[66]. This is important since *in vivo* and *in vitro* studies suggest that anti-angiogenic molecules may support embryogenesis. For example, there is decreased maternal blood concentration of soluble fms-like tyrosine kinase-1 (sFlt-1) in pregnant women who miscarry their pregnancy [67], [68], *Flt1* knockout mouse embryos die at GD8.5-9.0 due to excessive blood vessel growth [69]–[71], and the administration of pro-angiogenic vascular endothelial growth factor (VEGF) in early pregnancy leads to embryonic resorption in mice [72]. Since sFlt-1 acts as an inhibitor of angiogenesis [73], it has been suggested that blocking excessive VEGF signaling would lead to vascular hyperpermeability and the leak of serum proteins [71], [72], [74]. However, both the overactivation of anti-angiogenic pathways and inhibited placental angiogenesis in the second half of pregnancy have been identified as central to the pathogenesis of obstetrical syndromes [63]–[65], [74]–[117]. Among these, preeclampsia is characterized by increased placental expression and maternal systemic concentrations of anti-angiogenic sFlt-1 and soluble endoglin, which sequester circulating angiogenic factors, leading to an anti-angiogenic state, generalized endothelial dysfunction, hypertension and proteinuria [63]–[65], [74]–[81], [83]–[93], [95]–[98], [100], [102]–[106], [109]–[117].

Interestingly, besides the evolutionary conserved sFlt-1-i13 that is highly expressed in the mammalian placenta, three other sFlt-1 variants are also expressed in the human placenta. Among these, hsFlt-1-e15a has predominant placental expression among other tissues, and it is the most abundant placenta-expressed sFlt-1 variant in healthy pregnancies and those affected by preeclampsia [70], [71], [74], [118], [119]. These findings suggest that hsFlt-1-e15a may have important functions in normal pregnancy; however, its overexpression may promote the development of preeclampsia.

This latter effect was firstly proven by our parallel *in vivo* study that utilized the overexpression of this full-length hsFlt-1-e15a in mice [120]. Our results were in accord with those from a recent study that utilized the overexpression of the full-length hsFlt-1-i13, the second most abundant sFlt-1 variant in the human placenta and generated late-onset preeclampsia in mice [121]. These two *in vivo* studies demonstrated that the overexpression of either of these full-length human sFlt-1 variants is capable of the induction of the full spectrum of clinical symptoms of preeclampsia. This is of importance since most previous anti-angiogenic preeclampsia models utilized the overexpression of an artificially truncated mouse sFlt-1 mutant [msFlt-1(1-3)] [78], [90], [122]–[130], which lacks the highly conserved sFlt-1 domains important in dimerization, bioavailability and yet unknown functions [70], [131], and which may induce a stronger preeclampsia phenotype than the full-length mouse sFlt-1-i13 [131]. Our parallel *in vivo* study indirectly has also suggested that full-length hsFlt-1-e15a may have milder effects in inducing hypertension and proteinuria in mice than the truncated msFlt-1(1-3) [120].

Therefore, in the current study we aimed to: 1) compare the biological effects of the full-length hsFlt-1-e15a with that of the truncated msFlt-1(1-3) on the development of preeclampsia in mice; 2) examine the biological effects of these distinct sFlt-1 variants on the fetus, mother and placenta; and 3) investigate the tissue distribution of hsFlt-1-e15a viral transgene expression and its relation to the induced clinical symptoms.

## Materials and Methods

### Ethics statement

The animal study protocol (A#11-03-11) was approved by the Institutional Animal Care and Use Committee (IACUC) of Wayne State University (Detroit, MI). Animal handling and care followed all standards in strict accordance with the recommendations in the “Guide for the Care and Use of Laboratory Animals” of the National Institutes of Health [132]. All surgeries were performed under isoflurane anesthesia, and all efforts were made to minimize suffering. Adult mice were euthanized by CO_2_ inhalation on postpartum day 8 (PPD8), and offsprings were euthanized by decapitation immediately after delivery in accordance with the “Guidelines on Euthanasia” of the American Veterinary Medical Association and the IACUC guidelines at Wayne State University. Collection and utilization of human samples for research purposes were approved by the Institutional Review Boards of the *Eunice Kennedy Shriver* National Institute of Child Health and Human Development (NICHD), National Institutes of Health (NIH), Department of Health and Human Services (DHHS, Bethesda, MD, USA) and Wayne State University. Written informed consent was obtained from all pregnant women prior to the collection of clinical data and tissue samples. These specimens were coded, and data were stored anonymously.

### Animals and husbandry

**Figure 1:**
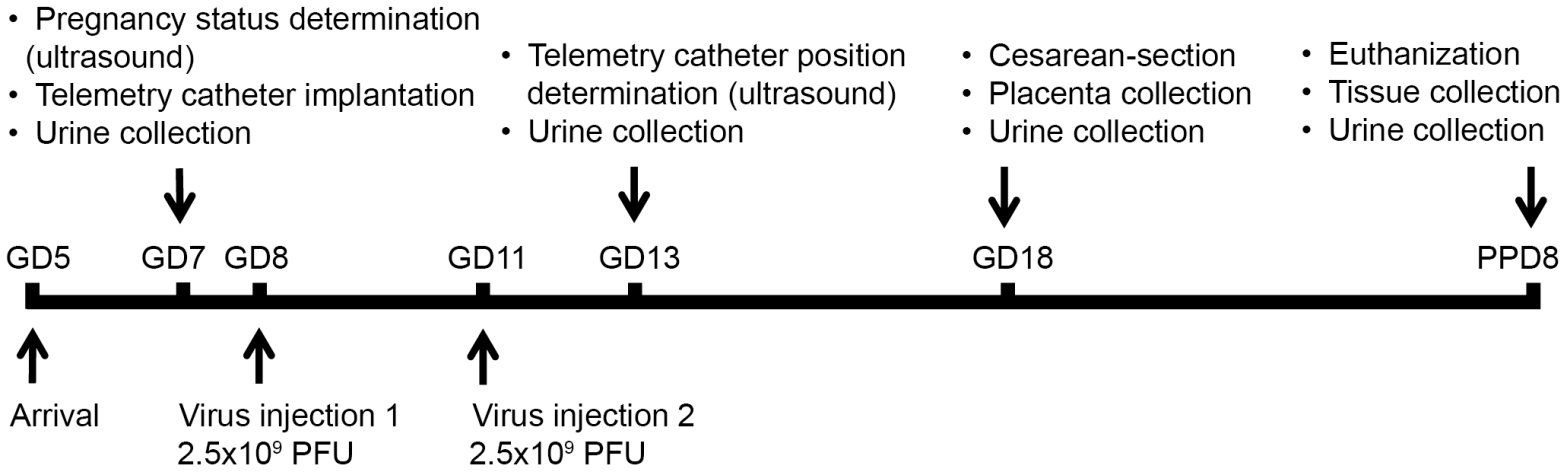
Experimental procedures. The flow-chart shows the experimental procedures performed at certain time-points during the study. GD, gestational day; PPD, postpartum day; PFU, plaque forming unit.

Timed-pregnant CD-1 mice (n = 48) were shipped on gestational day 5 (GD5) from Charles River Laboratories (Wilmington, MA, USA), and were housed separately under a 12-hour light/dark cycle, at constant temperature and humidity in the Division of Laboratory Animal Resources at Wayne State University. Mice were fed an *ad libitum* diet. Food and water intake, appearance, behavior and vital signs were monitored daily. Animals were excluded from the study in case of miscarriage, surgical complications, or any condition that a veterinarian deemed severe enough to warrant exclusion. **Figure 1** shows the experimental procedures performed at certain time-points during the study.

### Evaluation of pregnancy status with high-frequency ultrasound

Ultrasound scans were performed on GD7 to evaluate pregnancy status [120]. Anesthesia was induced with 4–5% isoflurane (Aerrane, Baxter Healthcare Corporation, Deerfield, IL, USA) and 1–2 L/min O_2_, and maintained with 2% isoflurane and 1–1.5 L/min O_2_. Mice were stabilized on a heating pad, and fur was shaved from the abdomen and neck areas. A 55 MHz ultrasound probe (Vevo 2010, Visual Sonics Inc., Toronto, ON, Canada) was used to scan for the signs of gestational sacs, advanced endometrial reaction and embryos.

### Telemetric blood-pressure catheter implantation and data acquisition

Immediately after ultrasound scans, mice with confirmed pregnancy underwent blood pressure catheter implantation under continued isoflurane anesthesia [120]. Briefly, mice were stabilized on a surgical platform that allowed for body temperature control. After a small vertical midline incision on the neck, the left common carotid artery was exposed and incised, and the catheter tip was positioned into the aortic arch. The telemetry transmitter (TA11PA-C10 or HD-X11, Data Sciences International, St. Paul, MI, USA) was placed in a subcutaneous pocket in the left flank. After skin closure, 2% lidocaine (Vedco Inc., St. Joseph, MO, USA), 0.5% bupivacaine (Hospira Inc., Lake Forest, IL, USA) and carprofen (5 mg/kg/24 h, Rymadil, Pfizer Inc., New York, NY, USA) were used to reduce postoperative pain. Body fluids were replenished by the injection of 0.5 ml of 0.9% sterile saline subcutaneously. Then mice were placed in cages supported by a warm water circulating blanket, and vital signs were regularly checked. Telemetric blood pressure monitoring on conscious, unrestrained mice started on GD11 and continued until PPD7 using the Dataquest A.R.T. 4.31 software (Data Sciences International). Telemetry catheter tip positions were verified with a 40 MHz linear ultrasound probe (Visual Sonics Inc.) on GD13.

### Adenoviral gene delivery

**Figure 2:**
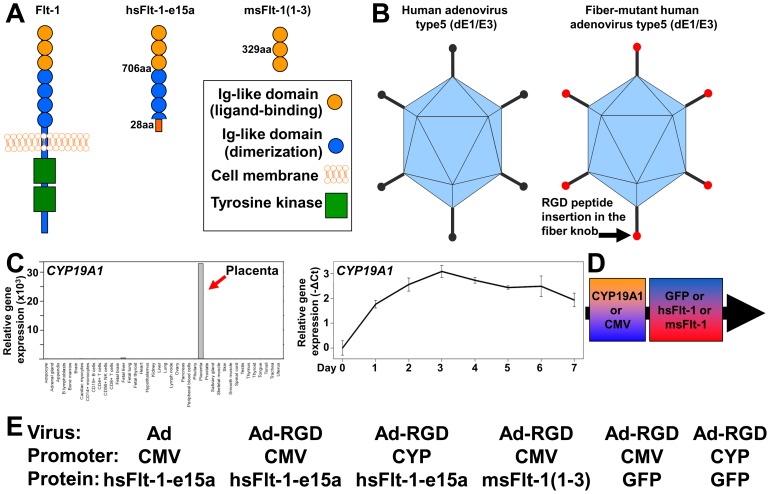
The development of viral constructs for the various treatment groups. (**A**) Human and mouse Flt-1 contains seven extracellular Ig-like domains and an intracellular tyrosine kinase, from which the first three Ig-like domains are important in ligand-binding, while the 4–7th Ig-like domains in receptor dimerization. The most abundant placental sFlt-1 variant in humans, sFlt-1-e15a, contains six Ig-like domains and a unique C-terminus encoded by exon 15a, which is located within a primate-specific AluSeq retrotransposon. The truncated mouse sFlt-1 mutant [msFlt-1(1-3)] contains only the first three Ig-like domains of Flt-1. (**B**) Besides the replication deficient human adenovirus Type5 (dE1/E3), “RGD fiber-mutant” adenoviruses were also used. (**C**) Besides the CMV promoter that has a strong promoter activity, the human *CYP19A1* promoter was also used. *CYP19A1* is strongly and predominantly expressed in the placenta among 40 human tissues (left), and its expression increases during trophoblast differentiation (right). Relative gene expressions are shown on the Y-axes. (**D–E**) Various combinations of viruses, promoters and transgenes used in this study.

In order to test the biological effects of differences in hsFlt-1-e15a expression patterns, we used three different viral vectors constructed from replication deficient adenovirus (Type5, dE1/E3) and an “RGD fiber-mutant” adenovirus with distinct tissue-tropism as well as two different gene promoters that differ in terms of tissue-specific promoter activity (**Figure 2**). The “RGD fiber-mutant” adenovirus that contains an RGD (Arg-Gly-Asp) motif on the fiber knob was developed in conjunction with Vector BioLabs (Philadelphia, PA, USA) according to that described by Mizuguchi et al., 2001 [133]. Adenoviruses and fiber-mutant adenoviruses expressing the full-length hsFlt-1-e15a, the truncated msFlt-1(1-3&^rpar; or green fluorescent protein (GFP) were constructed and titered by Vector BioLabs. HsFlt-1-e15a was overexpressed by 1) a wild-type adenovirus under the control of the cytomegalovirus promoter (Ad-CMV-hsFlt-1-e15a; n = 6), 2) an RGD fiber-mutant adenovirus under the control of the cytomegalovirus promoter (Ad-RGD-CMV-hsFlt-1-e15a; n = 6), or 3) an RGD fiber-mutant adenovirus under the control of the human *CYP19A1* promoter (Ad-RGD-CYP-hsFlt-1-e15a; n = 5). Truncated msFlt-1(1-3) was overexpressed by the RGD fiber-mutant adenovirus under the control of the cytomegalovirus promoter [Ad-RGD-CMV-msFlt-1(1-3); n = 6]. GFP was overexpressed by 1) the RGD fiber-mutant adenovirus under the control of the cytomegalovirus promoter (Ad-RGD-CMV-GFP; n = 12), or 2) by the RGD fiber-mutant adenovirus under the control of the human *CYP19A1* promoter (Ad-RGD-CYP-GFP; n = 4). According to the technique described by our parallel study [120], mice in these treatment and control groups were injected via the tail vein with 2.5×10^9^ plaque-forming units (PFU) of adenovirus constructs (in 100 µl saline) on GD8 and then repeatedly with 2.5×10^9^ PFU adenoviral constructs or saline on GD11 (**Figure 1**). A group of mice (n = 9) that was used only for the expression profiling of endogenous mouse transmembrane Flt-1 and sFlt-1-i13 received only 100 µl saline injection on GD8 and GD11 via the tail vein.

### Minimal invasive survival cesarean section

Aseptic survival cesarean section was developed by our parallel study and performed on GD18 [120]. Briefly, after pre- and intra-operative preparations and a 1–1.5 cm midline incision on the abdomen, short segments of the uterine horns were exteriorized separately, and fetuses and placentas were removed through 4 to 6 short incisions. Hysterectomies were closed with a single suture, the abdominal cavity was flushed with 0.9% saline solution, the muscular layer of the abdominal wall was closed with a continuous suture, and the skin was closed with staples. Local analgesia with 2% lidocaine and 0.5% bupivacaine adjacent to the incision site and subcutaneous carprofen was used for pain relief. Body fluids were replenished by injecting sterile saline subcutaneously, and postoperative care was similar to that following telemetric catheter implantation.

### Tissue collection

In accord with protocols described in our parallel publication [120], all fetuses were separated from the placenta and umbilical cord, and fetuses and placentas were weighted with a Scout Pro SP402 digital scale (Ohaus Corp., Pine Brook, NJ, USA) immediately after survival cesarean-section. The first four placentas adjacent to the cervix were collected from both horns of the uterus. The first placentas adjacent to the cervix were fixed in 4% paraformaldehyde (PFA) diluted with phosphate buffered saline (PBS, Gibco, Life Technologies Corporation, Grand Island, NY, USA) for 24 h, then dehydrated in 70% graded ethanol (Richard-Allan Scientific Dehydrant, Thermo Fisher Scientific Inc., Waltham, MA, USA), and embedded in paraffin. The second placentas were collected and homogenized in TRIzol (Invitrogen, Life Technologies Corporation, Carlsbad, CA, USA), and stored at −80°C until use. The third placentas were snap-frozen in liquid N_2_ and stored at −80°C until analysis. The fourth placentas were embedded into Tissue-Tek OCT Compound, snap-frozen in liquid N_2_, and stored at −80°C until analysis (Sakura Finetek USA, Inc., Torrance, CA, USA). On PPD8 after euthanization, tissues from the dams (spleen, uterus, liver, kidney and brain) were removed and cut into several pieces for 4% PFA fixation and paraffin embedding, homogenization in TRIzol reagent, or snap-freezing with liquid N_2_.

### Histopathological evaluation

Four-µm-thick sections were cut from paraffin embedded kidney blocks, mounted on silanized slides, deparaffinized and rehydrated. The general morphology was analyzed on selected tissue levels after staining with hematoxylin and eosin (H&E). Selected kidney sections were stained with periodic acid Schiff (PAS) reagent and with the Jones basement membrane reticulum stain (Dako Artisan Link Pro, Dako North America, Inc., Carpinteria, CA, USA) for the evaluation of the glomerular capillary loop basement membranes. Two pathologists (SJ and FQ) blinded to the clinical outcome evaluated 10 glomeruli from each kidney for glomerular endotheliosis (occlusion of glomerular capillaries, capillary tip ballooning, capillary tip swelling, and abnormalities of the capillary loop basement membranes) and changes in the mesangium. Glomerular damage was scored as follows: 0 = no glomerular changes in 10 glomeruli examined; 1+  = 1 to 5 of 10 glomeruli examined with either segmental or diffuse endotheliosis; 2+  = 6 or more glomeruli with segmental or diffuse endotheliosis. Images were taken with an Olympus BX50F light microscope (Olympus America Inc., Melville, NY, USA).

### Immunohistochemistry

Selected layers of placentas from control mice were immunostained for CD31 using a rabbit anti-CD31 monoclonal antibody (1∶50 dilution; Spring Bioscience, Pleasanton, CA, USA) and the Bond Polymer Refine Detection Kit (Leica Microsystems, Wetzlar, Germany) on a Leica Bond Max automatic staining system (Leica Microsystems).

### Aortic ring assays

Aortic ring assays were utilized to investigate endothelial functions *in vitro* after treatment with VEGF for six days [120]. Briefly, thoracic aortas were dissected under surgical microscope and placed into DMEM+GlutaMAX low glucose medium (Gibco, Life Technologies Corp.) containing Petri dishes. The ablation of periadventitial adipose tissue was followed by the sectioning of the thoracic aortas into 1 mm-long rings. For serum starvation, aortic rings were incubated in 12-well plates at 37°C in Opti-MEM+GlutaMAX reduced serum medium (Gibco, Life Technologies Corp.) overnight. Subsequently, aortic rings were placed into 96-well tissue culture plates pre-coated with 50 µl of Growth Factor Reduced BD Matrigel Matrix (BD Biosciences, Bedford, MA, USA) and then were covered with an additional 50 µl of Matrigel and 100 µl of Opti-MEM medium supplemented with 1% Penicillin–Streptomycin (Gibco, Life Technologies Corp.), 2.5% fetal bovine serum (FBS; Atlanta Biologicals, Lawrenceville, GA, USA), and 30 ng/ml of VEGF (ProSpec, East Brunswick, NJ, USA). After a six-day incubation at 37°C (with the medium changed every second day), aortic rings were fixed with 4% PFA diluted with PBS (Gibco, Life Technologies Corp.).

### Urine collection and albumin-creatinine immunoassays

As developed by a parallel study [134], urine samples were collected by ultrasound-guided cystocentesis on GD7, GD13, GD18 and PPD8. The Albuwell kit (Exocell Inc., Philadelphia, PA, USA) was used for murine urinary albumin determination, while the Creatinine Companion assay (Exocell Inc.) was used to measure creatinine [120].

### Primary human trophoblast isolation and cultures

Human placentas (n = 4) were collected from normal pregnant women who delivered a healthy neonate at term. Cytotrophoblasts were isolated by a method modified from Kliman et al. [135]. Briefly, villous tissues were cut into pieces, rinsed in PBS, and digested sequentially with Trypsin (0.25%; Invitrogen, Life Technologies Corp.) and DNAse I (60 U/ml; Sigma-Aldrich Corp. St. Louis, MO, USA) (90 min, 37°C). Dispersed cells were filtered through 100 µm Falcon nylon mesh cell strainers (BD Biosciences, San Jose, CA, USA), and then erythrocytes were lysed with NH_4_Cl (Stemcell Technologies, Vancouver, BC, Canada). Washed and resuspended cells were layered over Percoll gradients (20–50%) and centrifuged (20 min, 1200 g). The bands containing trophoblasts were collected; non-trophoblastic cells were excluded by negative selection with anti-CD9 (20 µg/ml) and anti-CD14 (20 µg/ml) mouse monoclonal antibodies (R&D Systems, Minneapolis, MN, USA) and MACS anti-mouse IgG microbeads (Miltenyi Biotec, Auburn, CA, USA). Trophoblasts were plated on collagen-coated plates (BD Biosciences; 5×10^6^ cells/well) in triplicate and kept in Iscove's modified Dulbecco's medium [Invitrogen, Life Technologies Corp.; supplemented with 10% fetal bovine serum (FBS), 5% human serum and 1% penicillin/streptomycin (P/S)] for 7 days. Cells were harvested for total RNA isolation in every 24 hours in triplicate.

### Total RNA isolation, cDNA generation, and quantitative real-time RT-PCR

Tissues were homogenized in TRIzol reagent, and total RNA was isolated using the QIAshredder (Qiagen, Valencia, CA, USA) and RNeasy Mini Kit (Qiagen). Total RNA was isolated from primary human trophoblast cultures with TRIzol reagent (Invitrogen) and RNeasy kit (Qiagen) according to the manufacturers' recommendations. Five hundred nanograms of total RNA was reverse transcribed with the SuperScript III First-Strand Synthesis System (Invitrogen). TaqMan assays (Applied Biosystems, Life Technologies Corp., Foster City, CA, USA) for human *FLT1* (Hs01052961_m1), mouse *Flt1* (Mm01210866_m1: exon boundary 1–2; Mm00438980_m1: exon boundary 15–16), *GFP* (Mr04097229_mr), human *CYP19A1* (Hs00903411_m1), as well as the endogenous human and mouse control genes [*RPLP0* (Hs99999902_m1); *Gapdh* (Mm99999915_g1)] were used for quantitative real-time RT-PCR performed on the Biomark System (Fluidigm, San Francisco, CA, USA) according to the manufacturer's recommendation.

### Confocal microscopy

Five-µm-thick tissue sections were cut from OCT-embedded snap-frozen placentas collected from Ad(RGD)-CMV-GFP and Ad(RGD)-CYP-GFP injected mice, and were mounted on silanized slides. Tissue sections were fixed with −20°C aceton for 10 min and then rinsed three times in ice-cold PBS. Tissue sections were then mounted with ProLong Gold Antifade Reagent and 4',6-diamidino-2-phenylindole (DAPI; Invitrogen, Life Technologies Corp.) and were imaged by a Leica TCS SP5 spectral confocal system (Leica Microsystems CMS GmbH, Mannheim, Germany) at the Microscopy, Imaging and Cytometry Resources Core of Wayne State University School of Medicine. Aortic rings, after fixation, were also mounted with ProLong Gold antifade reagent with DAPI, and image stacks were acquired on the same confocal microscope. To accommodate the size of the aortic rings without sacrificing resolution, 2×2 tiles were acquired using 20x magnification and an open pinhole.

### Data and statistical analyses

#### Gene expression profiling

Relative gene expression levels were quantified by averaging target (human *FLT1*, mouse *Flt1* or *GFP*) and reference (*Gapdh*) gene Ct values over technical replicates, and then subtracting the mean target gene Ct values from the mean reference gene Ct values within each sample. The expression values across different arrays were further adjusted using calibration samples. The Student's t-test was used to compare gene expression levels between treatments in a given tissue. In addition, mouse transmembrane Flt-1 expression was calculated from the data generated by the Mm00438980_m1 TaqMan assay, which is targeted to exon boundary 15-16, and thus, detects only the full-length Flt-1 mRNA expression levels. Mouse sFlt-1-i13 mRNA expression was calculated by subtracting full-length Flt-1 mRNA expression levels from the expression data generated by the Mm01210866_m1 TaqMan assay, which is targeted to exon boundary 1–2, and thus, detects all full-length and alternatively spliced Flt-1 mRNA levels. We used a linear model to estimate the effect of the transgene (either GFP or hsFlt-1-e15a) and the vector (either the CYP or CMV promoter groups or the two groups merged) on endogenous mouse sFlt-1-i13 expression.

#### Blood pressure

Mean arterial blood pressure (MAP) was calculated from systolic and diastolic blood pressure at each time point. MAP values for each mouse on a given day (GD or PPD) were averaged. Within the dataset of each mouse, the mean MAP value on GD11 was subtracted from all blood pressure data to obtain a normalized blood pressure, ΔMAP. A separate Linear Mixed Effects (LME) model was fit to the data for the time periods before and after cesarean delivery (GD18). The fixed effect terms in the model included the treatment group, polynomial terms of the gestation day (up to 3^rd^ and up to 2^nd^ degree for the periods before and after cesarean delivery, respectively), and their interaction terms. The random components in the mixed effects models included an intercept term and a quadratic term of gestational day for each animal. A likelihood ratio test comparing the fit quality of the model with and without interaction terms between the group and gestational day was used to test if the blood pressure profile over gestation was different between the groups. The blood pressure levels at specific gestational days were compared between the groups using a t-test.

#### Glomerular changes, urine albumin/creatinine ratios

Glomerular damage scores were evaluated using a logistic regression model. Albumin/creatinine ratios at different time points were compared with a Student's t-test.

#### Aortic ring assays

The angiogenic response of the aortic rings was analyzed by quantifying the microvessel outgrowth. A ruleset was developed using Definiens Developer XD2 (Definiens, Munich, Germany) to analyze the 3D confocal microscopy images. A series of segmentation and classification operations was performed on the DAPI channel to exclude the ring from the volume measurements, and the total volume of the objects determined to be “outgrowth” was summed for each image stack and reported. Volume data were averaged for the same ring, and then was further averaged over the multiple rings for the same animal. A t-test was used to compare the volume data between the groups.

#### Fetal survival rates, fetal and placental weights

The number of total and live fetuses, the fetal survival rate (live/total), the maternal weights, the total (as well as average) fetal and placental weights, and the total placental/total fetal weight ratios were compared between the treatment and control groups using t-tests.

#### Microarray and qRT-PCR data visualization

Microarray gene expression profiles for human tissues and cells were downloaded from the SymAtlas/BioGPS database [136], and expression data for 40 adult and fetal tissues was visualized via barplots using the R statistical environment (www.r-project.org) (**Figure 2C**). Primary human trophoblast *CYP19A1* expression data were normalized to the reference gene (*RPLP0*) obtained for each sample as -ΔCt_(*gene*)_ = Ct*_(RPLPO)_*-Ct*_(gene)_* and displayed as a function of time (**Figure 2C**).

## Results

### The development of various transgene delivery systems

In order to compare the effects of the full-length hsFlt-1-e15a with that of the truncated msFlt-1(1-3), viral constructs containing these two transgenes were constructed (**Figure 2A**). Since previous studies demonstrated that an “RGD fiber-mutant” adenovirus has a tissue tropism distinct from the replication deficient (Type5, dE1/E3) adenovirus [133], [137], we used both to investigate the effect of varying tissue expression profiles of hsFlt-1-e15a on its biological effects (**Figure 2B**). BioGPS data located the major and predominant expression of human *CYP19A1* to the placenta, and we observed that the expression of this gene strongly increased during villous trophoblast differentiation (**Figure 2C**). Since previous data showed that the 501 bp placenta-specific promoter of human *CYP19A1* is able to drive placenta-specific gene expression in transgenic mice [138], we tested the effect of this 501 bp *CYP19A1* promoter besides the CMV promoter in our viral constructs. **Figure 2D** and **Figure 2E** show the various combinations of viruses, promoters and transgenes used in this study.

### Unique placental expression of msFlt-1

**Figure 3:**
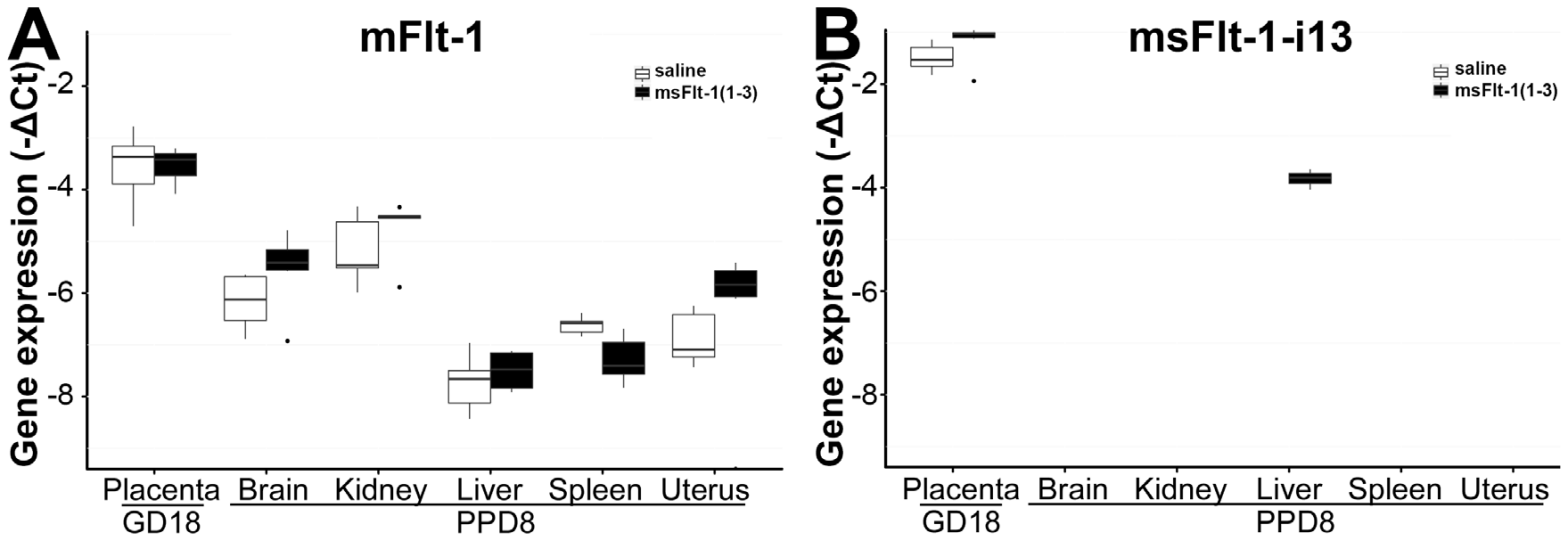
Profiling of mFlt-1 and msFlt-1-i13 expression. (**A**) Boxplots show the endogenous expression profile of the mouse transmembrane Flt-1 mRNA in placentas harvested on gestational day (GD) 18 and in five tissues harvested on postpartum day (PPD) 8. Endogenous Flt-1 mRNA expression was highest in the placenta in both the non-treated (saline) and the msFlt-1(1-3)-treated mice. (**B**) Boxplots show the endogenous expression profile of the mouse sFlt-1-i13 mRNA in placentas harvested on GD18 and in five tissues harvested on PPD8. Endogenous msFlt-1-i13 mRNA expression was restricted to the placenta in control animals, and transgenic msFlt-1(1-3) expression was detected in the placenta and the liver.

First, we aimed to detect the expression profile and levels of msFlt-1 in mice that had not received any viral injection. Total RNAs were isolated from placentas harvested on GD18, as well as from brain, kidney, liver, spleen, and uterine tissues harvested on PPD8. The expression of the endogenous transmembrane mFlt-1 mRNA was the highest in the placentas among the six tissues of mice that had not received virus injection (**Figure 3A**); msFlt-1-i13 mRNA expression was solely detected in the placentas of these non-treated animals (**Figure 3B**). Of note, the placental expression of msFlt-1-i13 mRNA was the highest among all tissues, genes and transcripts investigated in this study (**Figure 3B**). When we injected mice with the viral construct overexpressing the truncated msFlt-1(1-3), we observed the appearance of msFlt-1 mRNA expression in the liver (**Figure 3B**). The placental transcript levels of msFlt-1-i13 mRNA were not evidently increased in animals injected with the viral construct overexpressing truncated msFlt-1(1-3) compared to saline-treated mice, suggesting that the endogenous placental msFlt-1-i13 mRNA expression is higher than that of the transgene expressed msFlt-1(1-3) mRNA (**Figure 3B**).

### Expression patterns of various viral transgenes

**Figure 4:**
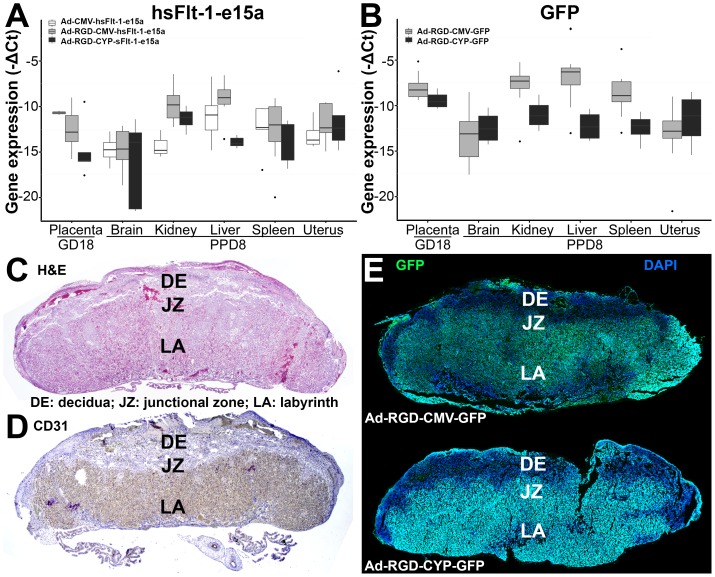
Profiling of hsFlt-1-e15a and GFP expression. (**A**) Boxplots show the expression profile of the transgenic hsFlt-1-e15a mRNA overexpressed by three different adenoviral vectors (Ad-CMV-hsFlt-1-e15a; Ad-RGD-CMV-hsFlt-1-e15a; Ad-RGD-CYP-hsFlt-1-e15a) in placentas harvested on gestational day (GD) 18 and in tissues harvested on postpartum day (PPD) 8. (**B**) Boxplots show the expression profile of GFP mRNA overexpressed by two different vectors (Ad-RGD-CMV-GFP; Ad-RGD-CYP-GFP) in placentas harvested on GD18 and in tissues harvested on PPD8. (**C**) Control placenta, H&E staining, 20x magnification. (**D**) Control placenta, anti-CD31 immunostaining, 20x magnification. The CD31 immunopositivity of the labyrinthine vessels are clearly seen. (**E, F**) Confocal microscopic images of placentas from GFP-treated mice. The placental expression of adenoviral GFP was the strongest in the labyrinth in both the Ad-RGD-CMV-GFP and Ad-RGD-CYP-GFP treated groups.

To compare the expression patterns of hsFlt-1-e15a and GFP, total RNAs were isolated from tissue samples harvested from virus-infected mice. Human hsFlt-1-e15a and GFP mRNA expression varied according to the viral constructs (adenovirus or fiber-mutant adenovirus) and promoters (CMV or CYP). The fiber-mutant adenovirus supported a higher hsFlt-1-e15a mRNA expression in the kidney and liver compared to the adenovirus, while the CYP promoter restricted hsFlt-1-e15a mRNA expression in the liver compared to the CMV promoter (**Figure 4A**). Similarly, the CYP promoter restricted GFP mRNA expression in the liver (49.8-fold down-regulation, p = 0.005), kidney (9.3-fold down-regulation, p = 0.02) and spleen (13.5-fold down-regulation, p = 0.01) compared to the CMV promoter, leading to the highest GFP mRNA expression in the placenta (**Figure 4B**). GFP expression was mainly restricted to the labyrinth zone of the placenta irrespective of the promoter in RGD fiber-mutant virus injected mice (**Figure 4C–E**).

Since it is important to know whether the transgene affects endogenous gene expression, we have tested the impact of the transgene and the vector on endogenous mouse sFlt-1-i13 expression. Importantly, we did not find any significant effect of either the transgene (GFP or hsFlt-1-e15a) or the vector (either the CYP or CMV promoter groups or the two groups merged) on the endogenous expression of msFlt-1-i13.

### Blood pressure telemetry monitoring

**Figure 5:**
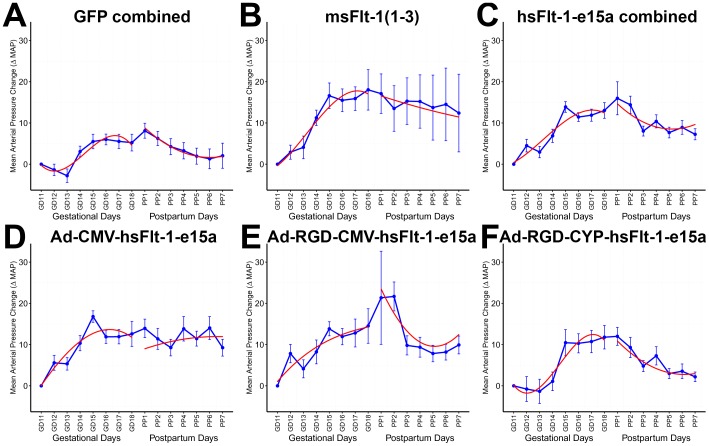
Blood pressure monitoring. X-axes show gestational days (GDs) and postpartum days (PPDs). Mean arterial pressure changes (ΔMAP) are depicted with blue curves. Blue lines show +/−standard errors. Red curves depict the ΔMAP patterns, fitted from the linear mixed effects model. (**A,B**) The blood pressure profile over gestation was different in msFlt-1(1-3)-treated mice from that in GFP-treated mice prior to cesarean delivery (p = 3.7×10^−5^; ΔMAP at parturition: 12.8 mmHg, p = 0.005). (**A,C**) The blood pressure profile over gestation in hsFlt-1-e15a-treated mice (all subgroups combined) was different from that in GFP-treated controls prior cesarean delivery (p = 4.3×10^−4^; ΔMAP at parturition: 7.8 mmHg, p = 0.009). (**D–F**) Among the three sub-groups of hsFlt-1-e15a-treated mice, those that received Ad-CMV-hsFlt-1-e15a and Ad-RGD-CMV-hsFlt-1-e15a had the highest increase in ΔMAP on GD15 (Ad-CMV-hsFlt-1-e15a: 11.3 mmHg, p = 0.0007; Ad-RGD-CMV-hsFlt-1-e15a: 8.3 mmHg, p = 0.009) and on GD18 (Ad-CMV-hsFlt-1-e15a: 7.4 mmHg, p = 0.09; Ad-RGD-CMV-hsFlt-1-e15a: 9.3 mmHg, p = 0.04) compared to controls. The blood pressure was 5 mmHg (GD15) and 6.6 mmHg (GD18) higher in Ad-RGD-CYP-hsFlt-1-e15a-treated mice than in control mice; however, p-values did not reach statistical significance (0.14 and 0.16, respectively).

The blood pressure profile over gestation was different in msFlt-1(1-3)-treated mice from that in GFP-treated mice (p = 3.7×10^-5^) prior to cesarean delivery. The ΔMAP at GD15 was 11.1 mmHg higher (p = 0.0008) in msFlt-1(1-3)-treated mice than in control mice, and this difference was even larger on GD18 (ΔMAP: 12.8 mmHg, p = 0.005) (**Figure 5A–B**). Of interest, one mouse in this group had a very high ΔMAP and a blood pressure of 175/135 mmHg on GD18. In contrast to all other mice in this group, this animal had constantly increasing blood pressure until PPD7 with a peak of 182/146 mmHg, resembling chronic hypertension following preeclampsia. The very high blood pressure values in this mouse skewed the mean MAPs in the postpartum period, causing the increased variance observed in **Figure 5B**.

Similarly, the blood pressure profile over gestation in hsFlt-1-e15a-treated mice (all subgroups combined: Ad-CMV, Ad-RGD-CMV, Ad-RGD-CYP) was different from that in GFP-treated controls (p = 4.3×10^−4^) prior to cesarean delivery. The ΔMAP at GD15 was 8.4 mmHg higher (p = 0.0005) in hsFlt-1-e15a-treated mice than in control mice, and it was 7.8 mmHg higher on GD18 (p = 0.009) (**Figure 5C**). Among the three sub-groups of hsFlt-1-e15a-treated mice, those that received Ad-CMV-hsFlt-1-e15a and Ad-RGD-CMV-hsFlt-1-e15a had the highest increase in ΔMAP on GD15 (Ad-CMV-hsFlt-1-e15a: 11.3 mmHg, p = 0.0007; Ad-RGD-CMV-hsFlt-1-e15a: 8.3 mmHg, p = 0.009) and on GD18 (Ad-CMV-hsFlt-1-e15a: 7.4 mmHg, p = 0.09; Ad-RGD-CMV-hsFlt-1-e15a: 9.3 mmHg, p = 0.04) compared to controls (**Figure 5D,E**). The blood pressure was 5 mmHg (GD15) and 6.6 mmHg (GD18) higher in Ad-RGD-CYP-hsFlt-1-e15a-treated mice than in control mice; however, p-values did not reach statistical significance (0.14 and 0.16, respectively) (**Figure 5F**).

### Morphological and functional changes in the kidneys in sFlt-1 treated mice

**Figure 6:**
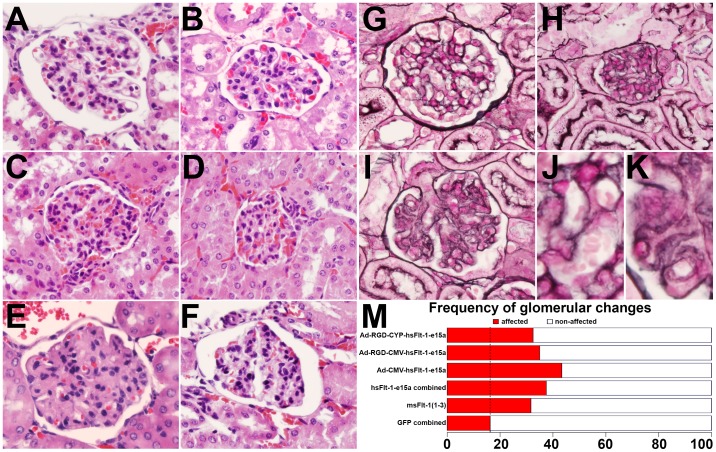
Histopathological evaluation of the kidneys. (**A,B,G**) Representative H&E (A: Ad-RGD-CMV-GFP, B: Ad-RGD-CYP-GFP) and Jones (G: Ad-RGD-CMV-GFP) stained sections show morphologically normal glomeruli in control animals. (**C,D,F,H**) Representative H&E (C: Ad-RGD-CMV-sFlt-1-e15a, D: Ad-RGD-CYP-sFlt-1-e15a, F: Ad-CMV-sFlt-1-e15a) and Jones (H: Ad-RGD-CMV-sFlt-1-e15a) stained sections show glomeruli with signs of swollen capillary endothelial cells and occlusion of glomerular capillaries in mice overexpressing sFlt-1-e15a. (**E,I**) Representative H&E (E) and Jones (I) stained sections show glomeruli with signs of swollen capillary endothelial cells and occlusion of glomerular capillaries in the dam overexpressing msFlt-1(1-3) with chronic hypertension. 400x magnifications. (**J**) High magnification image (1200x) from sub-image G shows a normal capillary structure. (**K**) High magnification image (1200x) from sub-image I shows thickened capillary loops. (**M**) The glomerular damage score was significantly higher in all treatment groups compared to the combined control group. Mice treated with msFlt-1(1-3) had an odds ratio (OR) of 2.4 for glomerular damage (p = 0.01). The OR for glomerular damage was 3.1 in hsFlt-1-e15a-treated mice (2.8×10^−5^). Among hsFlt-1-e15a-treated mice, mice in the Ad-CMV-hsFlt-1-e15a group had the largest OR (3.9, p = 4.8×10^−5^) for glomerular damage.

The kidneys from GFP-treated mice showed widely open capillary loops which had thin delicate walls, and no segmental thickening or hypercellularity was noted (**Figure 6A,B**). These findings were confirmed using the Jones basement membrane reticulum stain, in which the capillary basement membranes were thin and delicate, and no mesangial thickening was seen (**Figure 6G**). In contrast, the most consistent histopathological changes seen in the kidneys of mice overexpressing hsFlt-1-e15a or msFlt-1(1-3) were focal and segmental, with swollen capillary endothelial cells, occlusion of glomerular capillaries, and focal mesangial thickening (**Figure 6C–F**). Scattered glomeruli appeared sclerotic. Glomerular capillary changes were further confirmed by PAS staining and Jones basement membrane reticulum stain, which showed thickened capillary loops and focal expansion of the mesangium (**Figure 6H**). The dam in the msFlt-1(1-3)-treatment group with the constantly increasing blood pressure had dramatic changes in kidney histology, with extensive glomerular lesions seen in all glomeruli examined. These glomeruli appeared to be somewhat enlarged with marked thickening and expansion of the mesangium, and marked occlusion of capillaries and thickened capillary loops (**Figure 6E**). In this mouse, Jones basement membrane reticulum stain showed marked thickening and reduplication of the capillary loop basement membranes (**Figure 6I**). **Figure 6K** shows severe capillary loop damage with reduplication of the capillary loop in this mouse at high magnification. A normal capillary loop stained with Jones basement membrane reticulum stain is shown in **Figure 6J** at high magnification.

Histopathological evaluations revealed that the glomerular damage score was significantly higher in all treatment groups compared to the combined control group. Mice treated with msFlt-1(1-3) had an odds ratio (OR) of 2.4 for glomerular damage (p = 0.01). The OR for glomerular damage was 3.1 in hsFlt-1-e15a-treated mice (p = 2.8×10^−5^). Among hsFlt-1-e15a-treated mice, mice in the Ad-CMV-hsFlt-1-e15a group had the largest OR (3.9, p = 4.8×10^−5^) for glomerular damage (**Figure 6M**).

**Figure 7:**
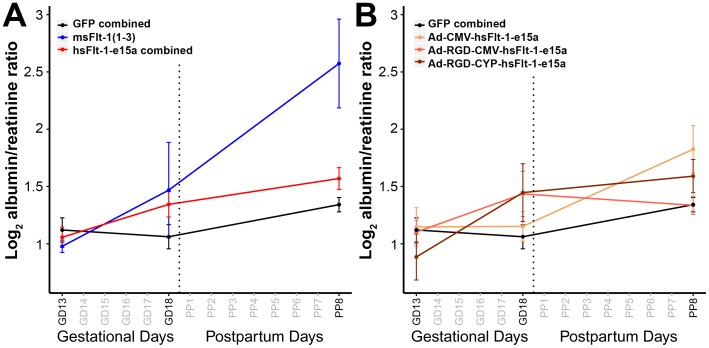
Functional evaluation of the kidneys. (**A**) Chart depicts albumin/creatinine ratios (in log scale) in urine specimens from mice in the GFP, msFlt-1(1-3) and hsFlt-1-e15a-treated groups. Mean urine albumin/creatinine ratios were higher in hsFlt-1-e15a-treated mice (GD18, p = 0.04 and PPD8, p = 0.03) and msFlt-1(1-3)-treated mice (PPD8, p = 4×10^−5^) than in controls. Proteinuria was extremely high in the mouse overexpressing msFlt-1(1-3) with chronic hypertension. (**B**) Subgroup analysis showed that hsFlt-1-e15a expressed under the CMV promoter in the adenovirus led to significant increase in albumin/creatinine ratio on PPD8 (p = 0.003); hsFlt-1-e15a expressed under the CMV promoter in the fiber-mutant adenovirus led to significant increase in albumin/creatinine ratio on GD18 (p = 0.04); while hsFlt-1-e15a expressed under the CYP promoter in the fiber-mutant adenovirus led to a marginally significant increase in albumin/creatinine ratio on GD18 (p = 0.056) and a significant increase on PPD8 (p = 0.04).

For functional evidence of kidney damage, we also determined albumin/creatinine ratios in urine samples obtained serially during pregnancy. As **Figure 7A** shows, mean urine albumin/creatinine ratios were higher in hsFlt-1-e15a treated mice (GD18, 1.9-fold, p = 0.04 and PPD8, 1.7-fold, p = 0.03) than in controls. The albumin/creatinine ratio was markedly elevated in msFlt-1(1-3)-treated mice in the postpartum period (17-fold, p = 4×10^−5^). The dam in the msFlt-1(1-3)-treatment group with the constantly increasing blood pressure had extreme proteinuria with albumin/creatinine ratios of 3,070 µg/mg on GD18 and 15,401 µg/mg on PPD8.

Subgroup analysis showed that Ad-CMV-hsFlt-1-e15a led to a significant increase in albumin/creatinine ratio on PPD8 (3-fold, p = 0.003); Ad-RGD-CMV-hsFlt-1-e15a led to significant increase in albumin/creatinine ratio on GD18 (2.4-fold, p = 0.04); while Ad-RGD-CYP-hsFlt-1-e15a led to a marginally significant increase in albumin/creatinine ratio on GD18 (2.4-fold, p = 0.056) and a significant increase on PPD8 (1.8-fold, p = 0.04) (**Figure 7B**). In summary, msFlt-1(1-3) had a stronger effect than hsFlt-1-e15a, and hsFlt-1-e15a expressed by the fiber-mutant adenovirus led to an earlier proteinuria than hsFlt-1-e15a expressed by the adenovirus.

### Aortic endothelial dysfunction caused by hsFlt-1-e15a and msFlt-1

**Figure 8:**
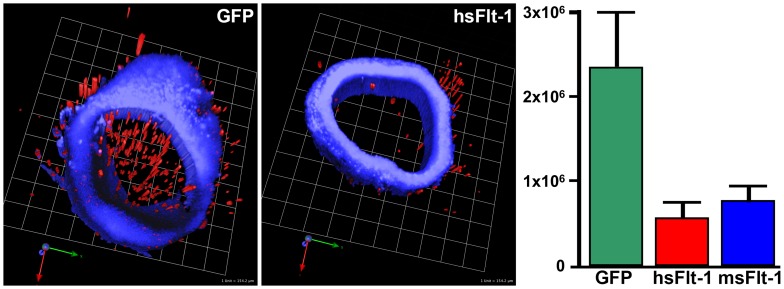
Aortic ring assays. Three dimensional reconstruction of confocal microscopic images of aortic rings from a mouse overexpressing GFP (left) or hsFlt-1-e15a (middle). Blue (4',6-diamidino-2-phenylindole, DAPI) depicts nuclei in the aortic rings, while red represents microvessel outgrowth volumes. The bar chart (right) depicts the differences in mean microvessel outgrowth volumes between the groups. Microvessel outgrowth from aortic rings was significantly decreased in hsFlt-1-e15a (p = 0.007) and msFlt-1(1-3)-treated (p = 0.02) mice compared to control animals.

We found that the mean microvessel outgrowth volume was 77% reduced in hsFlt-1-e15a overexpressing mice than in controls (p = 0.007), while the outgrowth volume was decreased by 66% in msFlt-1(1-3) overexpressing mice compared to controls (p = 0.02) (**Figure 8**). Of interest, in the msFlt-1(1-3)-treated dam with the constantly increasing blood pressure, the microvessel outgrowth volume was only 53% of the mean microvessel outgrowth volume in other mice in this group, showing a strongly dysfunctional endothelium.

### Human sFlt-1-e15a but not msFlt-1(1-3) increases litter sizes

**Figure 9:**
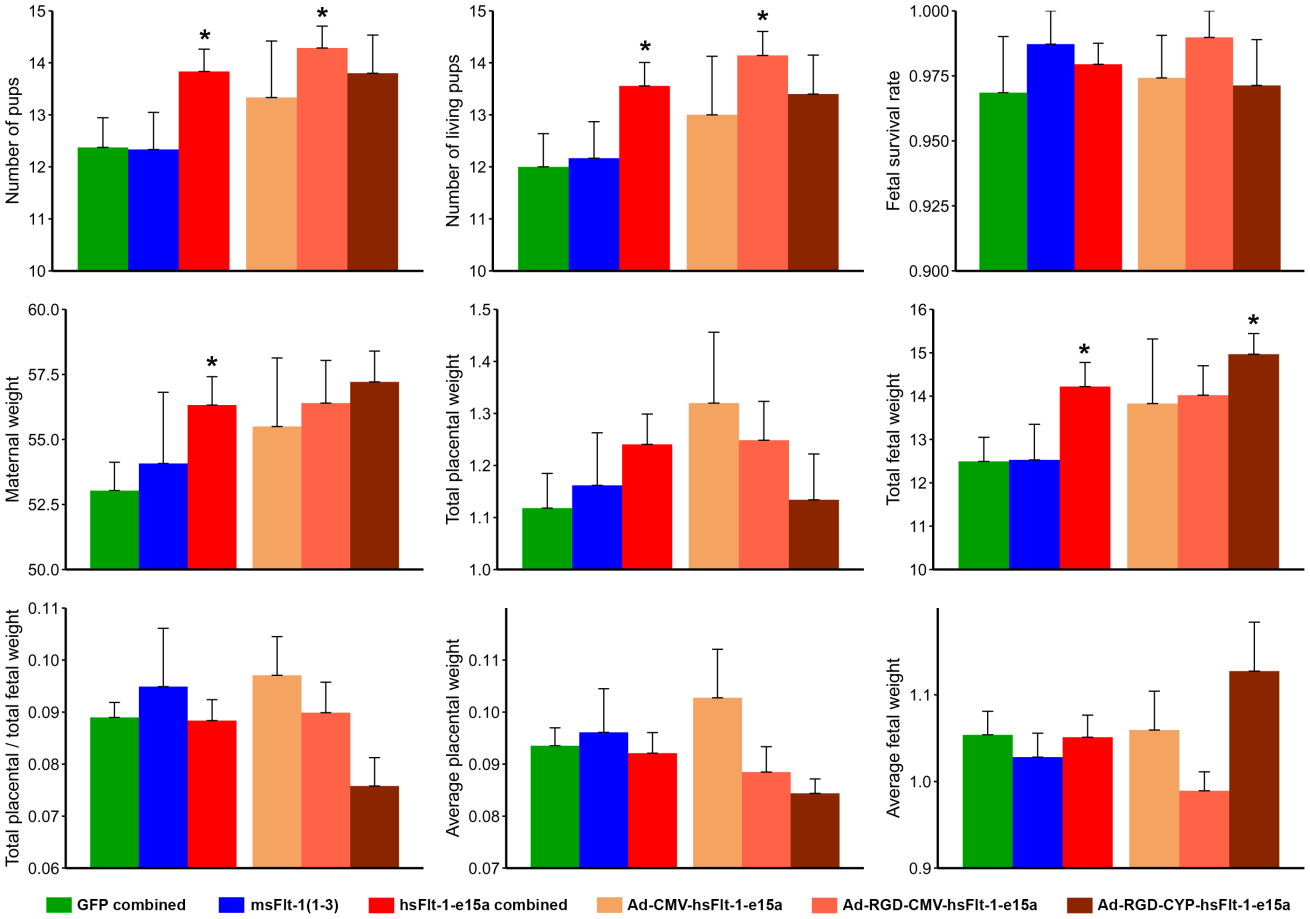
Litter sizes, maternal, placental and fetal weights, and placental/fetal weight ratios. Controls (n = 17) and msFlt-1(1-3)-treated mice (n = 6) had a litter size consistent with the strain average published by the vendor (n = 11.5). The number of pups (13.8±0.4, p = 0.046) and living pups (13.6±0.45, p = 0.05) were higher in hsFlt-1-e15a-treated mice (n = 18) than in controls. The total weight of living pups (14.2±0.56 g, p = 0.04) and maternal weights (56.3±1.1 g, p = 0.04) were also higher in hsFlt-1-e15a-treated mice than in controls. Truncated msFlt-1(1-3)-treated mice did not differ in any parameters from the controls. Among hsFlt-1-e15a-treated mice, the total weights of living pups was higher in the Ad-RGD-CYP-hsFlt-1-e15a treated mice than in controls (15±0.48 g, p = 0.043). The number of pups (14.3±0.42, p = 0.047) and the number of living pups (14.1±0.46, p = 0.039) was higher in the Ad-RGD-CMV-hsFlt-1-e15a treated mice than in controls. Stars denote statistical significance.

Fetal survival rate, average fetal weights, placental weights, and placental/fetal weight ratios were not affected by either hsFlt-1-e15a or msFlt-1(1-3) treatments (**Figure 9**). Controls (n = 17) and msFlt-1(1-3)-treated mice (n = 6) had a litter size consistent with the average published by the vendor (n = 11.5). The number of pups (13.8±0.4, p = 0.046) and living pups (13.6±0.45, p = 0.05) were higher in hsFlt-1-e15a-treated mice (n = 18) than in controls. The total weight of living pups (14.2±0.56g, p = 0.04) and maternal weights (56.3±1.1g, p = 0.04) were also higher in hsFlt-1-e15a-treated mice than in controls. Mouse sFlt-1(1-3)-treated mice did not differ in any parameters from the controls. When analyzing the subgroups of hsFlt-1-e15a-treated mice, the total weights of living pups was higher in the Ad-RGD-CYP-hsFlt-1-e15a treatment group than in controls (15±0.48g, p = 0.043). The number of pups (14.3±0.42, p = 0.047) and the number of living pups (14.1±0.46, p = 0.039) was higher in the Ad-RGD-CMV-hsFlt-1-e15a-treated mice than in controls.

## Discussion

### Principal findings of this study

This is the first study to compare the *in vivo* biological effects of full-length hsFlt-1-e15a, the most abundant sFlt-1 variant in the human placenta, and the truncated msFlt-1(1-3) that has been extensively used by anti-angiogenic models of preeclampsia in rodents. The principal findings of this study are as follows: 1) The full-length transmembrane mFlt-1 transcript is the most abundant in the placenta among six tissues investigated in mice; 2) The soluble mFlt-1-i13 variant is only expressed in the placenta in mice. Its unique and high level of expression suggest an important role for this soluble variant in the placenta; 3) The overexpression of the full-length hsFlt-1-e15a increased litter size, while the truncated msFlt-1(1-3) did not have this effect, underlining the important role of the full-length sFlt-1 in early pregnancy; 4) In the second half of pregnancy, both the full-length human and the truncated mouse sFlt-1 promoted the development of preeclampsia, including blood pressure elevation, glomerular damage, proteinuria, and endothelial dysfunction; 5) The truncated msFlt-1(1-3) also increased blood pressure and induced proteinuria similar to the full-length human sFlt-1-e15a; and 6) One dam with msFlt-1(1-3)-treatment had constantly high blood pressure, severe proteinuria and extensive glomerular damage, suggesting an increased sensitivity to sFlt-1 of this animal.

### The “good” face of sFlt-1 supports embryonic survival in early pregnancy

Angiogenesis is key to successful implantation, embryogenesis and placentation [60], [62], [70]-[72], [139], [140]. Among various molecule families that regulate placental angiogenesis, the gene family of VEGFs and placenta growth factor (PlGF) as well as their receptors have major regulatory roles [69]–[72], [139]. The placenta itself is a rich source of these molecules, synthesizing them in characteristically changing amounts during gestation [59], [62]–[66], [72], [139], [141]–[144]. The major cell-surface receptors of VEGF, Flt-1/VEGFR-1 and VEGFR-2, belong to a group of structurally similar receptors containing 7 Ig-like domains in their extracellular region, and an intracellular tyrosine kinase domain [70], [71], [145]. Interestingly, a soluble isoform of Flt-1, sFlt-1-i13, which lacks the tyrosine kinase domain but contains an evolutionary highly conserved unique amino-acid tail, is thought to have an important biological role [71]. Moreover, three other alternatively spliced sFlt-1 isoforms have also been described in the human placenta [118], [146], among which hsFlt-1-e15a is primate specific [147], [148]. Of note, these four sFlt-1 isoforms account for 95% of all *FLT1* transcripts in the human placenta in healthy, term pregnancies [118]. Among these, hsFlt-1-e15a is predominantly expressed in the placenta in humans, and it is the most abundant placental sFlt-1 isoform after the first trimester [74], [118], [146]–[148].

A growing body of evidence has shown that in early pregnancy VEGF is predominantly produced over PlGF in the gestational sac [139], [149]–[152], and VEGF promotes vascular endothelial cell proliferation, migration, survival, tube-formation, and branching angiogenesis *in vitro*, as well as vascular permeability *in vivo* [70], [71], [139], [153]. Since *Plgf* null mice are healthy with no signs of embryonic maldevelopment, PlGF was suggested to indirectly promote angiogenesis by displacing VEGF from VEGFR-1 and making it available for VEGFR-2 binding, or by generating PlGF/VEGF heterodimers that are able to activate VEGFR-1 or to induce VEGFR-1/VEGFR-2 dimerization [154]. Further supporting the pivotal role of VEGF and its receptors in embryonic vasculogenesis and angiogenesis during early pregnancy, the knockout of *Vegf* or *Vegfr2* genes in mice leads to impaired angiogenesis, while the *Flt1* knockout embryos die on GD8.5-9 due to excessive blood vessel growth [69]–[71]. These data together suggest that the major signal transducer for VEGF is VEGFR-2, while Flt-1 and its soluble isoforms strongly bind and neutralize VEGF, and thus have negative effect on angiogenesis and vascular permeability [70], [71], [73], [139]. Since the administration of VEGF in early pregnancy or endometrial overexpression lead to embryonic resorption in mice [72], [155], it has been suggested that the most important role of the Flt-1/sFlt-1 system is to maintain a barrier and block excessive VEGF signaling during embryogenesis that would lead to vascular hyperpermeability, the leak of serum proteins, and embryonic death [71], [72], [155].

In theory, our findings on the higher number of pups in hsFlt-1-e15a-treated mice may support this concept if hsFlt-1-e15a reduced the bioavailability of mouse VEGF. Alternatively, this rescue effect of hsFlt-1-e15a might have been the result of its interference with the mouse Flt-1/msFlt-1 system and the bioavailability of mouse PlGF. In our study, circulating mouse PlGF and VEGF concentrations could not be measured; thus, this question needs to be addressed in a future study. Nevertheless, hsFlt-1-e15a was shown to be a predominant placental VEGF-inhibiting protein by functional experiments [74]. Moreover, human sFlt-1-i13 was shown to bind and sequester mouse PlGF *in vivo* since the parallel overexpression of these molecules in mice led to decreased circulating hsFlt-1-i13 concentrations compared to hsFlt-1-i13 overexpression alone [121]. These functional data along with the high sequential and functional conservation of sFlt-1 from birds to humans [70], [71], [156] support the relevance of the biological actions of human sFlt-1 on the VEGF/PlGF/VEGF receptor system in mice. Importantly, our treatments started on GD8, exactly before the time-window (GD8.5-9) when *Flt1* knock-out embryos are lost, supporting the theory that the Flt-1/sFlt-1 system is important in embryonic development and the relevance of our findings on the biological effects of hsFlt-1-e15a treatment in early pregnancy in mice [69]–[71]. Since the same rescue-effect was not detected in the case of treatments with the truncated sFlt-1(1-3), the dimerization domain or the unique tail of hsFlt-1-e15a must play a key role in this rescue-effect, underlining the evolutionary conservation of these protein domains.

When looking at the different hsFlt-1-e15a treatment subgroups, an interesting association could be observed. The mean number of pups and living pups in a litter seemed to parallel hsFlt-1-e15a expression in the uterus (**Figures 4** **and** **9**). Moreover, the mean and total placental weights in a litter seem to parallel hsFlt-1-e15a expression in the placenta. These results suggest that the expression and the local availability of hsFlt-1-e15a in the uterus and placenta probably have a positive impact on decidual and placental angiogenesis, supporting successful embryonic and placental development in mice. In spite of the differences between human and mouse placentation [157]–[161], these results may be relevant to human pregnancy, as well. Indeed, decreased maternal blood concentrations of sFlt-1 in the first trimester are associated with miscarriages [67], [68], supporting the idea that the protective mechanisms of Flt-1/sFlt-1 may also similarly function in early human pregnancy.

### The “bad” face of sFlt-1 promotes the development of preeclampsia in human pregnancy

In spite of the rescue-effect of sFlt-1 in early pregnancy, the clinical manifestations of preeclampsia are associated with an imbalance in angiogenic and anti-angiogenic factors in the maternal circulation in the second half of the pregnancy [63]–[65], [74]–[81], [83]–[93], [95], [98], [100], [102]–[106], [109]–[117]. Histopathological investigations of the placenta and the placental bed revealed that a large portion of cases with preeclampsia, especially those with early-onset, can be characterized by the failure of trophoblast invasion and abnormal transformation of maternal spiral arteries, leading to the lack of low-resistance, dilated vessels pivotal for the continuous, adequate blood supply to the placenta [162]–[170]. These structural maldevelopments lead to an intermittent blood flow, and the consequent endoplasmic, nitrosative and oxidative stress of the placenta [54], [55], [171]–[173]. It has also been uncovered that the damaged placenta releases large amounts of syncytiotrophoblast debris, anti-angiogenic molecules and pro-inflammatory cytokines into the maternal circulation [74], [78], [83], [88]–[90], [102], [111], [174]–[183], which then induce an exaggerated maternal immune activation [28], [42], [174], [176], [184]–[186], generalized endothelial dysfunction, and damage of the kidneys [174], [176], [187]–[195]. Because of the extensive placental damage and loss of function, these early-onset cases of preeclampsia are frequently associated with intrauterine growth restriction [176], [196]–[200]. On the contrary, placental histopathological changes are less frequent in late-onset preeclampsia [178], [200]–[203], where fetal growth restriction occurs infrequently or even overgrowth of the fetus can be seen [198], [204]–[206]. It has recently been suggested that in these late-onset preeclampsia cases the overcrowding of the placental villi may cause placental stress, leading to the terminal pathway of preeclampsia [207].

This terminal pathway of preeclampsia, characterized by an imbalance of angiogenic and anti-angiogenic molecules in the maternal circulation, has been extensively investigated [59], [61], [63]–[65], [67], [74]–[81], [83]–[93], [95]–[98], [100], [102]–[106], [109], [111]–[117]. Longitudinal studies have shown that the increase in sFlt-1 and the decrease in PlGF concentrations precede the clinical onset of preeclampsia by several weeks [63]–[65], [80], [85], [88], [91], [104]. Some studies have provided evidence for the placental overexpression of sFlt-1 in preeclampsia, suggesting that the major source of circulating sFlt-1 is the placenta [74], [118], [119], [146], which would explain why the delivery of the placenta relieves the clinical symptoms of preeclampsia [102], [165], [173], [174], [208]–[213]. Since sFlt-1 is a decoy receptor for PlGF and VEGF, its increased concentrations in maternal blood decrease the bioavailability of these angiogenic factors, severely inhibiting placental angiogenesis and maternal endothelial functions in the kidneys and in the systemic circulation [59], [61], [63]–[65], [67], [74]–[81], [83]–[93], [95]–[98], [100], [103]–[106], [109]–[117], [173], [211], [214].

### The “bad” face of sFlt-1 promotes the development preeclampsia in animal models

Animal studies have provided *in vivo* experimental support for the above observations in humans. In most anti-angiogenic murine models of preeclampsia, replication deficient adenoviruses with major tropism in the liver were used to overexpress sFlt-1, and thus, dominant placental sFlt-1 expression could not be achieved [78], [126], [131], [215]–[217]. Interestingly, a couple of unrelated studies demonstrated that an “RGD fiber-mutant” adenovirus has a 10-100x higher placental tropism than the replication deficient adenovirus [133], [137]. In another study, the human *CYP19A1* promoter was shown to drive placenta-specific gene expression in transgenic mice [138]. For these reasons we chose different combinations of adeno- and “RGD fiber-mutant” adenoviruses as well as the common CMV promoter and the *CYP19A1* promoter to generate distinct tissue expression patterns of sFlt-1 (**Figure 2**). Because of the high conservation between various, full-length and truncated isoforms of sFlt-1 in humans and mice [70], [218] and the lack of immunoassays which could differentiate between these, we investigated the expression of these distinct isoforms at the RNA level.

In certain combinations we were able to reduce the expression of the transgene in the liver, and we could detect the highest expression of GFP in the placenta among all tissues. According to the GFP expression pattern, the “RGD fiber-mutant” adenovirus had a preference for the labyrinth zone of the placenta where most angiogenesis occurs. In spite of having the main expression of hsFlt-1-e15a and msFlt-1(1-3) in this zone, we could not observe any effect of the truncated msFlt-1(1-3) or the full-length hsFlt-1-e15a on fetal or placental growth either. Since a recent study that used lentiviral expression of sFlt-1 in the placenta could detect some effects of this decoy receptor on fetal growth [121], it might suggest that the local concentrations of sFlt-1 in the placenta expressed by the adenoviral delivery systems may not reach a certain level, which would severely affect placental angiogenesis. However, the level of expression of the natural mouse sFlt-1-i13 in the current study was much higher in the placenta than the transgene in control mice, which had normal fetal growth. This observation suggests that sFlt-1 itself cannot have such a negative impact on placental angiogenesis, which would severely affect fetal growth in mouse pregnancies. Indeed, a study that investigated the clinical effects of sFlt-1(1-3) and soluble endoglin in rats detected that the overexpression of sFlt-1(1-3) itself was not able to decrease birth-weights, only when coupled with the overexpression of soluble endoglin [90]. Indeed, most of the studies in rodents recapitulated these results by not detecting decreased birth-weights in rodents overexpressing solely sFlt-1 [78], [90], [122]–[130]. Since our longitudinal study on human subjects previously demonstrated that sFlt-1 overexpression is a feature of both term and preterm preeclampsia, while soluble endoglin is overexpressed in cases of IUGR all along pregnancy and also in preterm preeclampsia [64], it is possible that soluble endoglin overexpression in parallel with that of sFlt-1 is a feature needed for the development of early-onset, IUGR-associated preeclampsia, if maternal or environmental factors otherwise do not exaggerate the phenotype of preeclampsia. This would mean that the phenotype of preeclampsia may depend on the angiogenic/anti-angiogenic molecule profile, which could be an interesting subject of later studies.

Of note, the plateaus in mean arterial blood pressures in both the msFlt-1 and hsFlt-1-treated animals occurred between GD15-18, seven to eight days after the adenovirus injections on GD8 and GD11. Since transient transgene expression by adenoviruses and the virus load decrease 7-10 days after virus injection [216], [217], [219], consistent with blood pressure peaks 8-10 days after injection of adenoviruses overexpressing sFlt-1 in rodents [122], [123], [126], [131], our results suggest that blood pressure plateaus occurred in parallel with the highest sFlt-1 transgene expression. The decrease in blood pressures was probably not only the result of the decrease in sFlt-1 transgene expression due to decreasing viral load, but also because of the delivery of the placentas expressing a considerable amount of sFlt-1. This is consistent with the findings in humans, where the delivery of the placenta, which produces the increased amounts of sFlt-1 variants almost exclusively [118], quickly decreases blood pressures and cures the clinical symptoms in preeclampsia [199], [200]. Similarly, the extracorporeal removal of sFlt-1 from maternal blood leads to the decrease in blood pressures and the improvement of the clinical condition in women with preeclampsia [220].

Interestingly, the truncated msFlt-1(1-3) had a similar effect on blood pressure elevation and proteinuria to the full-length hsFlt-1-e15a. Since the mouse placenta expresses levels of endogenous sFlt-1-i13 as high as in transgenic msFlt-1(1-3), the question occurred why this endogenous molecule did not cause severe preeclampsia-like symptoms in untreated animals. As has been discussed in previous publications [70], [218], the truncated sFlt-1(1-3), which lacks three IgG-like domains and a conserved tail region compared to the hsFlt-1-e15a variant, may have different bioavailability, circulating half-life, and angiogenic factor sequestering capability than the full-length sFlt-1. Indeed, evidence from other studies suggests a milder effect of the overexpressed full-length mouse sFlt-1 compared to the truncated msFlt-1(1-3) on kidney functions [120], [121], [131].

### The “ugly” face of sFlt-1 promotes the development of chronic disease following preeclampsia

Preeclampsia does not only affect pregnant women and their offspring during pregnancy, but it also has long-term consequences on their morbidity and mortality later in life. Epidemiologic studies have revealed that preeclampsia confers an increased risk of cardiovascular and cerebrovascular complications, such as chronic hypertension, ischemic heart disease, or stroke [214], [221]–[225]: 1) There is a two-fold risk of developing cerebrovascular or cardiovascular disease in women who had preeclampsia compared to normal pregnant women; 2) Twenty percent of women may develop cardiovascular disease within seven years of their pregnancy affected by preeclampsia; 3) Women with preeclampsia have a 2 to 10-fold risk of developing chronic hypertension postpartum than normal pregnant women; 4) Women with early-onset severe preeclampsia complicated by IUGR are at high risk of adverse long-term cardiovascular outcomes and an 8-fold increased risk of cardiovascular death compared to normal pregnant women; 5) Pregnant women with preeclampsia who delivered a low birth-weight neonate have an increased risk of chronic kidney disease later in life. Whether these long-term adverse outcomes result from the endothelial dysfunction and/or vascular damage in preeclampsia, or they point to pre-existing risk factors shared by preeclampsia and cardiovascular disease (chronic hypertension, diabetes, dyslipidaemia, hypercoagulability, insulin resistance, metabolic syndrome, obesity, etc.) is still a topic of debate.

In this regard it was relevant to observe that mice in the hsFlt-1-e15a and msFlt-1(1-3) treatment groups had endothelial dysfunction one week after delivery as shown by the aortic ring assays, suggesting long-term maternal systemic vascular effects of sFlt-1 overexpression during pregnancy. Moreover, one mouse in the msFlt-1(1-3) treatment group had a very high blood pressure elevation, which was still increasing after one week postpartum. This mouse also had dramatic glomerular damage, extremely high proteinuria, and the most dysfunctional aortic endothelium among all mice in the study group. These results suggest that this dam developed chronic cardiovascular and kidney disease as a result of preeclampsia. In a similar mouse preeclampsia model in another study, animals that received sFlt-1 treatment during pregnancy had characteristic changes in their plasma proteome six months after delivery with enrichment of proteins associated with cardiovascular and metabolic diseases, as well as inflammatory response [226]. That study and the current observation support the notion that some long-term adverse outcomes associated with preeclampsia may be the consequence rather than an underlying predisposition of this syndrome.

### Conclusions

Truncated msFlt-1(1-3) simulated the preeclampsia-promoting effects of full-length hsFlt-1 released from the placenta. Truncated mouse sFlt-1(1-3) had a strong effect on maternal endothelium but not on placentas and embryos. In contrast, full-length hsFlt-1-e15a induced symptoms of preeclampsia; however, it also increased litter size. In accord with the predominant placental expression of hsFlt-1-e15a and msFlt-1-i13, full-length sFlt-1 may have a role in the regulation of embryonic development. These observations point to the difference in the biological effects of full-length and truncated sFlt-1 and the changes in the effect of full-length sFlt-1 during pregnancy. Our results may also have important implications in the clinical management of preeclampsia.
